# Advances and Frontiers in Single‐Walled Carbon Nanotube Electronics

**DOI:** 10.1002/advs.202102860

**Published:** 2021-10-23

**Authors:** Jianping Zou, Qing Zhang

**Affiliations:** ^1^ Centre for Micro‐ & Nano‐Electronics School of Electrical and Electronic Engineering Nanyang Technological University Singapore 639798 Singapore

**Keywords:** carbon nanotubes, electronic devices, field‐effect transistors, integrated circuits, low‐power, subthreshold swing

## Abstract

Single‐walled carbon nanotubes (SWCNTs) have been considered as one of the most promising electronic materials for the next‐generation electronics in the more Moore era. Sub‐10 nm SWCNT‐field effect transistors (FETs) have been realized with several performances exceeding those of Si‐based FETs at the same feature size. Several industrial initiatives have attempted to implement SWCNT electronics in integrated circuit (IC) chips. Here, the recent advances in SWCNT electronics are reviewed from in‐depth understanding of the fundamental electronic structures, the carrier transport mechanisms, and the metal/SWCNT contact properties. In particular, the subthreshold switching properties are highlighted for low‐power, energy‐efficient device operations. State‐of‐the‐art low‐power SWCNT‐based electronics and the key strategies to realize low‐voltage and low‐power operations are outlined. Finally, the essential challenges and prospects from the material preparation, device fabrication, and large‐scale ICs integration for future SWCNT‐based electronics are foregrounded.

## Graphical abstract

Single‐walled carbon nanotube (SWCNT) electronics are shown to be capable of maintaining advances in computing power and energy efficiency in the more Moore era. Further development of SWCNT electronics for next‐generation high‐performance and low‐power integrated circuits (ICs) is still facing many daunting challenges from the material preparation, device fabrication, and circuit design and device–circuit system optimization.

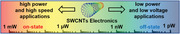

## 1 Introduction

As the dimension of electronic devices shrinks continuously along Moore's law,^[^ ^1^ ^]^ the physical limits to silicon (Si)‐based complementary metal–oxide–semiconductor (CMOS) transistors scaling have been reached, resulting in severe performance degradations due to the short‐channel effects,^[^ ^2^ ^]^ gate oxide tunneling, and unmanageable power consumption.^[^ ^3^, ^4^ ^]^ To further downscale the transistors without degrading their performance along the more Moore trend, great efforts have been devoted to exploring revolutionary new materials and device structures such as strained Si, nanotubes and nanowires, III–V materials, high‐*k* (dielectric constant) gate dielectric, as well as trigate device configuration.^[^ ^5^, ^6^, ^7^, ^8^, ^9^, ^10^ ^]^

Among these emerging electronic materials, single‐walled carbon nanotube (SWCNT) is the most promising electronic material, owing to its quasi‐1D sp^2^ structure, extremely high carrier mobility, thermal conductivity, and superior flexibility and stability.^[^ ^11^, ^12^, ^13^, ^14^, ^15^, ^16^ ^]^ Due to the excellent electrostatic gate control on the SWCNT channel,^[^ ^17^, ^18^, ^19^ ^]^ SWCNT‐field effect transistors (FETs) are expected to play an important role in the next‐generation digital integrated circuits (ICs) as the fundamental building blocks. Since report of the first SWCNT‐FET in 1998,^[^ ^20^ ^]^ a remarkable progress in SWCNT‐based electronics has been made, especially in the past decade. To study the transport properties and explore the excellent device performance with unique functionalities, many innovative device structures and process approaches have been adopted.^[^ ^19^, ^21^, ^22^, ^23^, ^24^, ^25^, ^26^, ^27^, ^28^, ^29^, ^30^, ^31^, ^32^, ^33^, ^34^, ^35^, ^36^, ^37^, ^38^ ^]^ The electrical performances of these devices have even outperformed Si‐based counterparts in comparable sizes. High‐performance individual SWCNT CMOS‐FETs with the gate length less than 10 nm have been demonstrated.^[^ ^30^ ^]^ These ultrasmall‐scaled SWCNT devices exhibited higher intrinsic carrier mobility and on‐state performance than Si CMOS‐FETs with a similar gate length but at a lower supply voltage. Furthermore, graphene‐contacted SWCNT‐FETs with a gate length of 5 nm have also been reported, where the conventional metal source (S) and drain (D) electrodes (e.g., Pd or Sc) were replaced with graphene.^[^ ^30^ ^]^ Because of broadening of the depletion region at the graphene/SWCNT contacts, the direct tunneling between the S and D is prohibited, leading to an essentially improved off‐state performance with a much smaller subthreshold swing (SS) of 73 mV dec^−1^. Based on low‐resistance end‐bonded contacts formed through a solid‐state reaction between a semiconducting SWCNT (s‐SWCNT) and deposited Co–Mo alloy S/D electrodes,^[^ ^39^ ^]^ a p‐channel SWCNT transistor with all components restricted within 40 nm has been constructed.^[^ ^31^ ^]^ The end‐bonded contact scheme allows the scaling of the contact length down to 10 nm without increasing the contact resistance. As a result, the sub‐10 nm gate length SWCNT‐FET can deliver a significantly higher current density above 0.9 mA µm^−1^ at a low supply voltage of 0.5 V with an SS of 85 mV dec^−1^. This remarkable performance has well exceeded that of mainstream Si devices.

Despite these notable achievements, the inabilities to precisely control specific chirality, diameter, position, and orientation of SWCNTs significantly increase the device‐to‐device variations and restrict realistic technology applications based on individual SWCNTs. In contrast, a SWCNT thin film that is composed of a huge number of individual SWCNTs, in the form of either random networks or aligned arrays, is immune to the challenges of individual SWCNTs because the thin film structure offers attractive homogenous electrical properties even with heterogeneous SWCNTs. This could also offer a large active area to achieve a high current driving capability.^[^ ^40^, ^41^, ^42^, ^43^ ^]^ A variety of electronic devices and circuits based on SWCNT thin‐film‐transistors (TFTs), such as the driver circuits for flat‐panel displays,^[^ ^44^, ^45^ ^]^ biosensors,^[^ ^46^, ^47^ ^]^ fundamental logic gates,^[^ ^43^, ^48^, ^49^, ^50^, ^51^, ^52^, ^53^ ^]^ medium‐ and large‐scale ICs,^[^ ^41^, ^54^, ^55^ ^]^ and even a 16‐bit microprocessor (RV16X‐NANO) composed of more than 14 000 CMOS SWCNT‐TFTs,^[^ ^56^ ^]^ have been demonstrated with excellent electrical performances and industrialization feasibility. However, several key issues are to be addressed in order for practical applications of SWCNT thin films as an active channel material in FETs, including the high packing density, high semiconducting purity, and high alignment of SWCNTs.^[^ ^57^, ^58^ ^]^ As‐produced SWCNTs are a mixture of semiconducting (s‐) and metallic (m‐) SWCNTs,^[^ ^59^, ^60^ ^]^ and the existence of m‐SWCNTs in a transistor channel could short the S and D electrically, leading to device failure. Great efforts have been devoted to selective synthesis of high purity s‐SWCNTs. One way is to synthesize s‐SWCNTs by particular catalyst design^[^ ^61^, ^62^ ^]^ or introduce a process of in situ selectively etching m‐SWCNTs during CNT growth based on the differences in reactivity and conductivity of s‐SWCNTs and m‐SWCNTs.^[^ ^63^, ^64^, ^65^, ^66^ ^]^ The other way is to sort SWCNTs by noncovalent functionalized post‐treatment, in which m‐ or s‐SWCNTs are selectively wrapped with specific surfactants/polymers. The commonly used noncovalent methods include density gradient ultracentrifugation,^[^ ^67^, ^68^ ^]^ chromatography,^[^ ^69^ ^]^ DNA,^[^ ^70^ ^]^ and conjugated polymer wrapping.^[^ ^60^, ^71^ ^]^ The s‐SWCNTs sorted by these methods usually possess extremely high purity up to 99.99%.^[^ ^59^, ^72^, ^73^, ^74^ ^]^ In addition to high semiconducting purity, a highly aligned array with high packing density is also required to implement large‐scale SWCNT‐based ICs. Compared with chemical vapor deposition (CVD)‐grown aligned SWCNTs, solution‐processed aligned SWCNTs exhibit higher packing densities and higher yields.^[^ ^58^, ^75^, ^76^, ^77^, ^78^ ^]^ All these advancements and achievements on the growth, sorting, and processing enable potential applications of SWCNT‐TFTs on ultralarge‐scale ICs. Recently, methylation‐induced reversible metallic‐semiconducting transition of CVD‐grown SWCNT arrays has been reported.^[^ ^79^ ^]^ It was found that selective reaction of methyl radicals to the surface of m‐SWCNTs could reversibly destroy *π*‐conjugated electronic structure and open up the bandgap of the methylation‐treated m‐SWCNTs, yielding a high purity of s‐SWCNTs (>97.5%). In addition, solution‐processed well‐aligned s‐SWCNT arrays with a purity of 99.9999% have been prepared on a 4 in. Si wafer.^[^ ^58^ ^]^ These aligned s‐SWCNT arrays have high packing density (between 100 and 200 tubes µm^−1^) with full surface coverage. Top‐gated transistors fabricated on these aligned s‐SWCNT arrays exhibited a high on‐state current exceeding 1.3 mA µm^−1^ and a peak transconductance of 0.9 mS µm^−1^ under a power supply of 1 V, which is better than that of commercial Si MOSFETs. Through modifications and optimizations of standard solution‐based processing methods, fabrication of SWCNT‐TFTs on industry‐standard 200 mm wafers using commercial Si manufacturing facilities has also been reported.^[^ ^80^ ^]^ The wafer‐scale uniformity and reproducibility of the device performances enable the SWCNT‐TFT technologies from research laboratories to commercial Si CMOS compatible manufacturing facilities.

Further development of SWCNT‐based transistors for next‐generation high‐performance and low‐power consumption ICs is still facing many daunting challenges from the material preparation, device fabrication, and circuit design and device–circuit system optimization. In this review, recent advances and breakthroughs in SWCNT‐based FETs/TFTs and their electronic applications will be the focus. We begin with the fundamental electronic structures and properties of SWCNTs in Section 2. Then, several device structures of SWCNT‐FETs will be introduced and discussed with the contact properties and the subthreshold switching properties of SWCNT‐FETs in Section 3. Section 4 explores the ballistic transport in SWCNTs and outlines the progress of SWCNT‐FETs/TFTs in high‐performance electronic applications. In Section 5, we concentrate on the state of the art low‐power SWCNT‐FETs/TFTs and the representative strategies to realize low‐voltage and low‐power operations. Finally, the key challenges and outlooks for SWCNT‐based high‐performance and low‐power electronics are summarized.

## 2 Structure and Electronic Properties of SWCNTs

**Figure 1:**
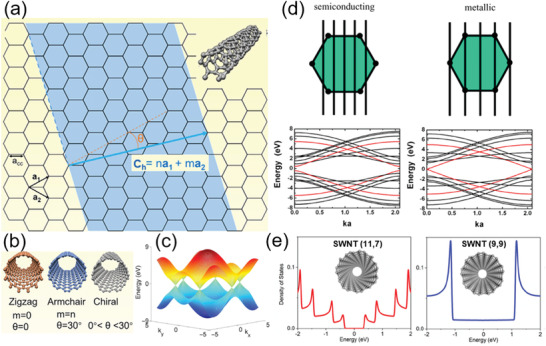
Atomic structure and electronic properties of SWCNTs. a) An individual SWCNT rolled up from a graphene sheet along a chiral vector *C*_h_. b) Classification of SWCNTs: zigzag, armchair, and chiral CNTs, respectively. (a,b) Reproduced with permission.^[^ ^82^ ^]^ Copyright 2020, Elsevier. c) The calculated energy dispersion contours of graphene. d) The band‐structures of SWCNTs with different electronic properties depending on the relation of the quantized lines to the Brillouin zone of graphene. (c,d) Reproduced with permission.^[^ ^89^ ^]^ Copyright 2006, IOP Publishing. e) The density of states (DOS) for (11, 7) and (9, 9) SWCNTs, respectively. Reproduced (Adapted) with permission.^[^ ^98^ ^]^ Copyright 2020, American Chemical Society.

An individual SWCNT can be structurally described as a seamless, hollow cylinder rolled up from a piece of graphene sheet along a chiral vector ***C***_h_ (**Figure** **1**a).^[^ ^81^, ^82^ ^]^ Most of SWCNTs have a diameter less than 2 nm, making them 1D nanostructure due to the high aspect ratio (i.e., the length over diameter). The chiral vector ***C***_h_ indicates the rolling‐up direction and defines the circumference of the SWCNT. Based on the basis vectors (***a***_1_ and ***a***_2_) of the graphene sheet, the vector ***C***_h_ can be defined as

(1) $$C_{h}=na_{1}+ma_{2}$$

where *n* and *m* are integers, and *n* ≥ *m*, $|a_{1}\left|=\right|a_{2}|=a=\sqrt{3}a_{C-C}$, *a*_C − C_ = 0.142 nm is the nearest‐neighbor C—C bond length. Thus, each SWCNT can be described by a pair of chiral indices (*n*, *m*) with a diameter $d_{\mathrm{CNT}}=\left|C_{h}\right|/\pi =\left(a/\pi \right)\;\sqrt{n^{2}+m^{2}+nm}$ and a chiral angle *θ* (the angle between ***C***_h_ and ***a***_1_).^[^ ^83^, ^84^, ^85^ ^]^ As shown in Figure 1b, in terms of the chiral indices and the chiral angle, SWCNTs can be categorized as zigzag tubes (*m* = 0, *θ* = 0°), whose chiral vectors are purely along ***a***_1_ or ***a***_2_, armchair tubes (*n* = *m*, *θ* = 30°), whose chiral vectors are along the direction exactly between ***a***_1_ and ***a***_2_, and chiral tubes (*n* ≠ *m*, 0° < |*θ*| < 30°).^[^ ^82^, ^86^ ^]^

Carbon atoms in SWCNTs are in the covalent sp^2^ hybridization, the same as those carbon atoms in graphene.^[^ ^87^, ^88^ ^]^ Thus, the electronic properties and the band structures of an SWCNT can be simply derived from that of graphene. Figure 1c shows the calculated energy dispersion contours of graphene using a tight‐binding model consisting only of *π* orbital electrons,^[^ ^89^ ^]^ where the upper π* antibonding band (conduction band) and the lower *π* bonding band (valence band) touch at six points (Dirac points) at the corners of the Brillouin zone.^[^ ^89^, ^90^ ^]^ The Fermi level passes through the six points, suggesting graphene is a zero‐bandgap semiconductor or semimetal.^[^ ^19^, ^87^, ^89^ ^]^ As electron motion is along not only the axis of an SWCNT, but also its circumference, the band structure of an SWCNT can be achieved by imposing a periodic boundary condition around the circumference of the nanotube.^[^ ^91^, ^92^ ^]^ Electron motion along the nanotube axis is free and the corresponding component of the momentum ***k***_||_ is continuous. In contrast, the perpendicular component of the momentum ***k***_⊥_ is along the direction of the chiral vector ***C***_h_ and quantized due to the periodic boundary condition

(2) $$k_{⊥}\cdot C_{h}=2\pi p$$

where *p* is a nonzero integer. Therefore, the allowed electronic states for an SWNCT are defined only by a set of parallel lines in the *k*‐space with quantized ***k***_⊥_ states superimposing on the 2D Brillouin zone of graphene (Figure 1d).^[^ ^83^, ^89^ ^]^ The 1D sub‐band structures of an SWCNT can be obtained via cross‐sectional cutting of the energy dispersion of graphene with these parallel quantized lines.^[^ ^84^, ^89^, ^90^ ^]^ The band structure in the vicinity of the Fermi level is determined by those ***k***_⊥_ states that are near the Dirac points and they govern the electrical transport properties in SWCNTs. If the quantized lines cut across the Dirac points of graphene (see the upper‐right panel in Figure 1d), the SWCNT is a metallic tube with a zero‐energy gap (see the lower‐right panel in Figure 1d). Otherwise, the SWCNT is a semiconducting tube with a finite energy gap between the valence and conduction bands (see the upper‐left and lower‐left panel in Figure 1d). In summary, an (*n*, *m*) SWCNT is semiconducting if *n* − *m* is not dividable by 3.^[^ ^90^, ^91^, ^92^, ^93^, ^94^ ^]^ The bandgap is inversely proportional to its diameter, i.e., *E*_g_ ≈ 0.7 eV/*d*_CNT_ (nm).^[^ ^95^ ^]^ Otherwise, the SWCNT is metallic with a relation of *n* − *m* = 3*q*, where *q* is an integer. All armchair (*n* = *m*) SWCNTs are metallic. However, owing to the curvature effect induced by small diameters, the rest of metallic (*n* ≠ *m*) SWCNTs exhibit quasi‐metallic behavior with a small bandgap (*E*_g_ ≈ *k*_B_ *T*) at room temperature.^[^ ^90^, ^96^, ^97^ ^]^

Figure 1e shows the density of states (DOS) for (11, 7) and (9, 9) SWCNTs, respectively.^[^ ^98^ ^]^ For the (11, 7) SWCNT, the DOS vanish, and an energy gap of ≈0.57 eV occurs at the Fermi level (*E* = 0), showing it is a semiconductor. In contrast, the DOS are finite in the vicinity of the Fermi level in the (9, 9) SWCNT, indicating a metallic tube. Due to the 1D characteristics, the 1D DOS of SWCNTs shows Van Hove singularities at higher energies.^[^ ^99^ ^]^

In addition to the unique electronic properties, SWCNTs also possess excellent mechanical properties resulting from the covalent sp^2^ bonding between carbon atoms. Their superior mechanical properties, e.g., high Young's modulus (270–950 GPa) and tensile strength (11–63 GPa),^[^ ^100^ ^]^ have made SWCNT a promising material for flexible and stretchable electronics.^[^ ^101^ ^]^

## 3 SWCNT‐FET Fundamentals

### 3.1 Device Structures of SWCNT‐FETs

**Figure 2:**
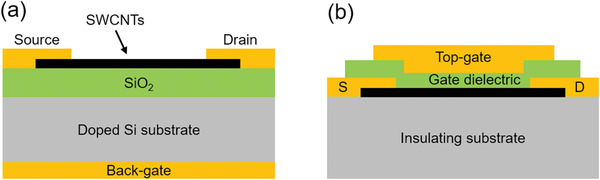
Schematic device structures of SWCNT‐FETs. a) A back‐gated device. b) A top‐gated device.

Since the first FET based on an individual SWCNT was demonstrated in 1998,^[^ ^20^ ^]^ several kinds of device structures of SWCNT‐FETs have been proposed and implemented. The two most common structures are back‐gated structure (**Figure** **2**a) and top‐gated structure (Figure 2b). In the back‐gated device, a heavily doped Si is normally used as the substrate and the back‐gate. The active channel consists of either an individual SWCNT or an SWCNTs thin film, separated from the gate using a thin dielectric layer. Although the back‐gated devices are easily fabricated and have been successfully employed for the purpose of proof‐of‐concept, they are not easily integrated with other components because of their global back‐gates. In contrast, the top‐gated structures are more suitable for device integration and have been extensively employed in SWCNT‐based ICs. The top‐gated devices are conventionally built on an insulating substrate, like a quartz wafer, a flexible plastic film, or a Si wafer with thermally grown SiO_2_. To obtain high gate capacitance and gate control efficiency, high‐*k* HfO_2_, ZrO_2_, and Y_2_O_3_ thin films have been adopted as the gate dielectric layers.^[^ ^9^, ^102^, ^103^ ^]^

**Figure 3:**
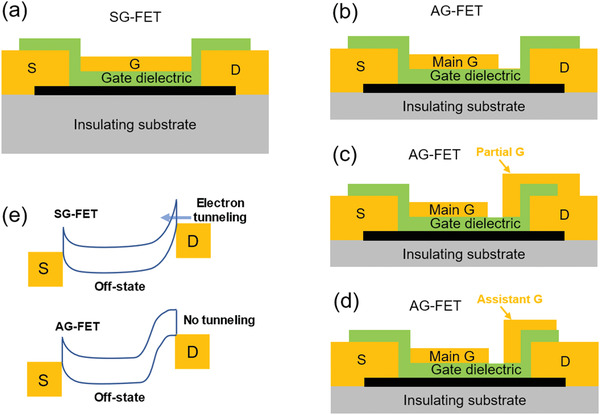
Schematic gate structures of SWCNT‐FETs. a) A self‐aligned symmetric‐gate FET (SG‐FET). b) Asymmetric‐gate FET (AG‐FET) with a gap between the G and the D electrodes. c) A dual‐gate FET with a partial‐gate connected to the D electrode. d) A dual‐gate FET with a specially biased assistant G near the D electrode. e) Schematic energy band diagrams (off‐state) of an SG‐FET and an AG‐FET at a high *V*_DS_.

In Si CMOS technology, a self‐aligned device structure is usually adopted to accurately position the edges of the S/D and the gate (G) electrodes so that there is no significant overlap or gap between these electrodes.^[^ ^104^ ^]^ This symmetric‐gate FET (SG‐FET) structure (**Figure** **3**a) has been employed for making high‐performance SWCNT‐FETs because of the minimized parasitic capacitances and leakage current between the G and S/D electrodes.^[^ ^24^, ^27^, ^54^ ^]^ In addition to the SG‐FET structure, asymmetric‐gate FET (AG‐FET) structures have been also proposed and realized for SWCNT‐FETs.^[^ ^105^, ^106^, ^107^, ^108^, ^109^, ^110^, ^111^ ^]^ In the AG‐FETs, the main‐gate covers most of the SWCNTs channel with a gap between the G and the D electrodes (Figure 3b). In addition, a partial‐gate connected to the D electrode (Figure 3c), or a specially biased assistant‐gate near the D electrode (Figure 3d) can be introduced. In the SG‐FETs (upper panel of Figure 3e), the barrier near the drain contact is very thin at the off‐state due to the strong gate control on the ultrathin body of the SWCNTs channel, giving rise to obvious electron tunneling current and ambipolar behavior. Contrarily, in the AG‐FETs, because of the gap between the G and D electrodes, the partial‐gate or a specially biased assistant‐gate near the D electrode, the main‐gate control on the energy band of the channel near the drain contact is significantly reduced, leading to a relative thick barrier near the drain contact (lower panel of Figure 3e), which could significantly suppress electron tunneling current and ambipolar behavior even at a high source–drain bias, *V*_DS_. **Table** **1** lists several kinds of device structures of SWCNT‐FETs and their advantages.

**Table 1:** Several kinds of device structures of SWCNT‐FETs and their advantages. (SG: symmetric gate; AG: asymmetric gate; BG: back‐gated; TG: top‐gated; SA‐TG: self‐aligned top‐gated)

	Device structure	Advantages of the device structures
SG‐FET	BG device (Figure 2a)	1) Simple and easy fabrication processes. 2) Suitable for purpose of proof‐of‐concept.
	TG device (Figure 2b)	1) High‐performance arising from highly efficient gate control capability through ultrathin high‐*k* gate dielectric. 2) Suitable for large‐scale device integration.
	SA‐TG device (Figure 3a)	High‐performance resulting from the reduced parasitic capacitances and leakage current between the G and S/D electrodes.
AG‐FET	AG with a gap between the G and D electrodes (Figure 3b)	High‐performance acheived from: 1) Suppression of off‐state leakage current. 2) Suppression of ambipolar behavior at high source–drain bias.
	A partial‐gate connected to the D electrode (Figure 3c)	
	A specially biased assistant G near the D electrode (Figure 3d)	

### 3.2 Contacts Properties of SWCNTs and Metal Electrodes

In a conventional Si MOSFET, the S and D electrodes are usually heavily doped Si, making the S (or D)/Si‐channel contacts ohmic. However, owing to the lack of simple and efficient doping approaches for SWCNTs, the S and D electrodes in a SWCNT‐FET are metals and the S (or D)/SWCNTs contact exhibits typical Schottky contact properties.^[^ ^15^, ^112^, ^113^, ^114^ ^]^ The Schottky barriers (SBs) at the metal electrodes/s‐SWCNT interface plays a significant role in the operation of SWCNT‐FETs.

When a metal is in contact with a bulk semiconductor such as silicon, metal‐induced gap states (MIGSs) form on the semiconductor surface. The MIGS decay exponentially away from the metal/semiconductor interface. Thus, a dipole sheet is created at the interface due to the charges in the semiconductor side and the image charges induced in the metal side. It results in bending of the energy band near the semiconductor surface. Thus, the position of the Fermi level (i.e., the charge neutrality level) at the semiconductor surface is pinned due to the MIGS.^[^ ^115^ ^]^ The Fermi level pinning effect dominates the formation of the Schottky barriers at the metal/bulk semiconductor interface, leading to nearly constant barrier height, which is only weakly dependent of the metal work‐function. However, the MIGS induced Fermi level pinning plays a minor effect in the metal/SWCNT contact due to the 1D ultrathin SWCNT.^[^ ^112^, ^116^, ^117^ ^]^ Self‐consistent calculations show that the MIGS in SWCNT decay more rapidly than in bulk semiconductors. As a result, the barrier width at the metal/SWCNT interface is only a few nanometers so that electrons or holes can effectively transport across the contacts, with a negligible effect of the Fermi level pinning. As a consequence, a metal/SWCNT contact is strongly affected by the work‐function difference between the metal and SWCNT.^[^ ^89^ ^]^ For example, a high work‐function metal, such as Pd (5.1 eV), easily forms a nearly transparent barrier (or a negative barrier) with an SWCNT since the Fermi level of the metal tends to line up with the valence band of the SWCNT, in favor of holes transport, but not for electrons transport. Contrarily, a low work‐function metal, such as Sc (3.3 eV), in contact with a SWCNT produces an almost transparent barrier for electrons, promoting electrons transport. If the electron barrier is much higher than the hole barrier, a SWCNT‐FET works as a p‐type FET. Otherwise, it operates as an n‐type FET. For the SWCNT with a small bandgap, its electron barrier is comparable with its hole barrier, the SWCNT‐FET is likely ambipolar.^[^ ^113^, ^114^, ^118^ ^]^

**Figure 4:**
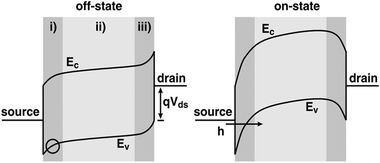
Energy band diagram of a Schottky‐contact SWCNT‐FET showing the carrier injection mechanism at the off‐state and on‐state, respectively. Reproduced with permission.^[^ ^15^ ^]^ Copyright 2002, American Physical Society.

As the band bending near the contacts of a SWCNT‐FET can be modulated by the applied gate voltage to achieve the control of the channel current, the device operates more like a SB transistor (**Figure** **4**).^[^ ^15^ ^]^ When the FET is at the off‐state, the SB at the source contact is thick and the tunneling current is very small. The off‐state current is mainly determined by the thermionic emission over the SB. With increasing of the gate voltage above the threshold voltage, *V*_TH_, the energy band of the SWCNT in the middle of the channel is raised gradually, leading to a very thin SB at the source contact and an increased tunneling current. The FET operates at the on‐state. Consequently, the carrier transport in a Schottky‐contact SWCNT‐FET is determined by the competition between the thermionic emission and thermally assisted tunneling, which is controlled by the gate voltage modulation of the SB at the source contact.^[^ ^119^ ^]^ This carrier transport mechanism is entirely different from that in conventional bulk‐switching transistors where the current is controlled by the thermionic emission over the bulk barrier inside the channel.^[^ ^15^ ^]^

The SB at a metal/SWCNT contact is easily affected by adsorbed oxygen.^[^ ^114^, ^120^, ^121^ ^]^ Typical SWCNT‐FETs built from as‐grown nanotubes are usually unipolar p‐type devices. It was observed experimentally that the adsorption of oxygen may change the work‐function of the metal and thus affect the SB and the device performance as well.^[^ ^114^ ^]^ After oxygen exposure, the conduction behavior of the device could change from n‐type to p‐type, suggesting that oxygen adsorption enhances hole conduction, but inhibits electron conduction.

### 3.3 Switching Properties in SWCNT‐FETs

Continuously scaling down of the feature size of CMOS FETs has enabled remarkable improvements in the integrated device density, circuit switching speed, functionality, and manufacturing cost of digital ICs. However, high power consumption becomes a bottleneck for further scaling down of ICs owing to the increasing leakage currents and difficulty in scaling down of the supply voltage, *V*_DD_,^[^ ^122^, ^123^ ^]^ and heat dissipation. In modern CMOS technology, the *V*_DD_ has been reduced to below 0.7 V.^[^ ^124^ ^]^ The difficulty of further scaling down of *V*_DD_ mainly arises from the limit (≈60 mV dec^−1^) of SS at room temperature.^[^ ^123^, ^125^, ^126^ ^]^

In terms of the operation modes of MOSFETs, when the gate bias (*V*_GS_) is greater than the threshold voltage (*V*_TH_), the device operates in the above‐threshold region, in which the on‐state current increases almost linearly with the bias voltage *V*_GS_. The variation of the drain current (*I*_DS_) with *V*_GS_ can be characterized by transconductance, *g*_m_, which is defined as

(3) $$g_{m}=dI_{\mathrm{DS}}/dV_{\mathrm{GS}}\;$$

A higher *g*_m_ could offer higher current driving capability and signal amplifying capability of the device in the above‐threshold region. When *V*_GS_ < *V*_TH_, the device works in the subthreshold region. The subthreshold switching behavior is generally characterized by SS, which is defined as the inverse of the subthreshold slope

(4) $$\mathrm{SS}\;={\left(d\log_{10}\left(I_{\mathrm{DS}}\right)/dV_{\mathrm{GS}}\right)}^{-1}$$

The SS reflects the required gate voltage that causes the variation of the subthreshold current by one order of magnitude. Therefore, a small SS implies a steep switching behavior of the device, enabling a low off‐state leakage current and thus a minimized passive power.^[^ ^127^ ^]^ The SS of SWCNT‐FETs can be empirically obtained from the transfer characteristic of the device. It can be derived from electronic transport analysis based on carrier thermionic emission theory^[^ ^128^ ^]^

(5) $$\mathrm{SS}\;=\frac{dV_{\mathrm{GS}}}{d\log_{10}\left(I_{\mathrm{DS}}\right)}\;\approx \frac{k_{B}T}{e}\left(1+\frac{C_{\mathrm{it}}}{C_{\mathrm{ox}}}\right)\ln10$$

where *k*_B_ *T* is the thermal energy; *e* is electron charge; *C*_it_ is the capacitance induced by interface trap states and *C*_ox_ is the gate dielectric capacitance. The *C*_ox_ in SWCNT‐FETs can be analytically expressed as^[^ ^129^ ^]^

(6) $$C_{\mathrm{ox}}={\left\{\frac{1}{2\pi \varepsilon_{0}\varepsilon_{r}}\ln\left[\frac{\Lambda_{0}}{\pi R_{T}}\sinh\left(\frac{2\pi t_{d}}{\Lambda_{0}}\right)\right]+C_{q}^{-1}\right\}}^{-1}\;\Lambda_{0}^{-1}$$

where *ε*_0_ *ε*_r_ is the dielectric constant of the gate dielectric; Λ_0_ is the average distance between the tubes; *R*_T_ is the tube radius; *t*_d_ is the thickness of the gate dielectric and *C*_q_ (≈10^−10^ F m^−1^)^[^ ^130^ ^]^ is the intrinsic quantum capacitance of SWCNTs.

According to the above expressions of SS and *C*_ox_, lowering the trapped charge density (i.e., reducing *C*_it_) at the SWCNTs‐channel/gate dielectric interface, and increasing *C*_ox_ via reducing the gate dielectric thickness *t*_d_, or using high‐*k* dielectric materials are the most straightforward and effective approaches to reduce the SS or, in other word, increase the gate control capability. For an ideal case, *C*_it_ ≪ *C*_ox_, the SS could reach its limit ((*k*_B_ *T*/*e*)ln10≅60 mV dec^−1^) at room temperature. This fundamental limit of the SS is mainly determined by the temperature‐dependent thermionic emission of carriers over an energy barrier.^[^ ^115^, ^131^ ^]^

**Figure 5:**
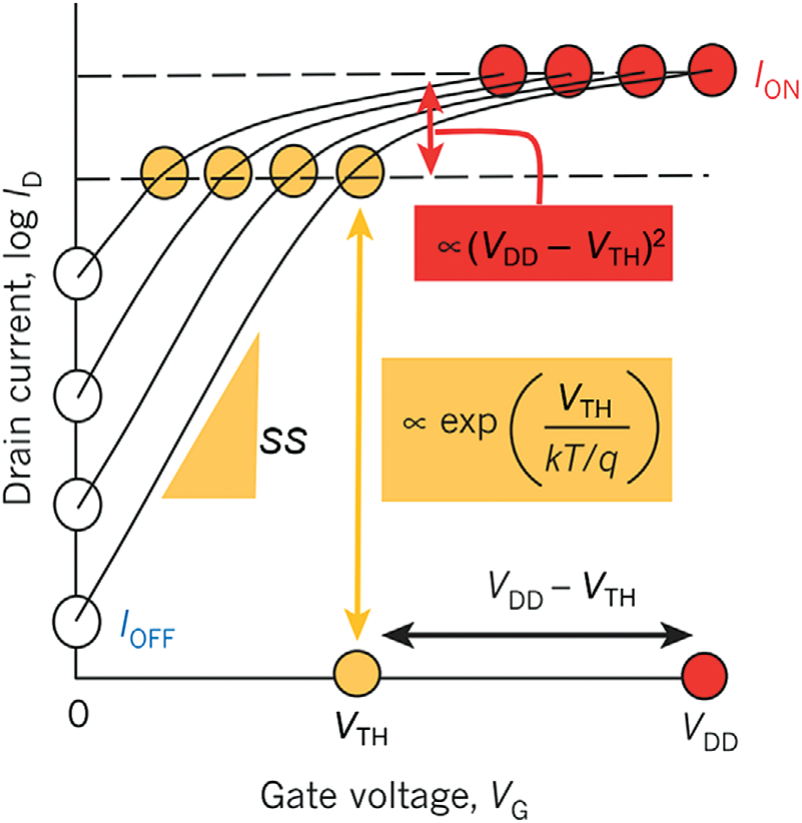
Schematic transfer characteristics of a conventional MOSFET with scaling down of *V*_DD_ and *V*_TH_. Due to the unscalable subthreshold swing SS at room temperature, the off‐state current *I*_OFF_ increases exponentially. Reproduced with permission.^[^ ^123^ ^]^ Copyright 2011, Springer Nature.

The power consumption of digital ICs is usually calculated by the formula *P* = *V*_DD_ × *I*_DD_. Therefore, reducing the supply voltage *V*_DD_ together with a low off‐state leakage current is very important to achieve a low power dissipation. Downscaling of *V*_DD_ requires a simultaneously lowered *V*_TH_ so that the on‐state performance (*I*_ON_) can be maintained via keeping the overdrive factor (*V*_DD_ − *V*_TH_) constant. As a result, the off‐state current (*I*_OFF_) inevitably increases exponentially (see the intersections between the current–voltage (*I*_D_ − *V*_G_) curves and the vertical axis in **Figure** **5**) because of an unscalable SS with a limit of 60 mV dec^−1^ at room temperature.^[^ ^123^ ^]^ Therefore, further downscaling of *V*_DD_ < 0.5 V, while maintaining a reasonable on/off current ratio, *I*_ON_/*I*_OFF_ ≥ 10^4^ and a lower power consumption, requires a different carrier injection approach (e.g., band‐to‐band (BTB) tunneling^[^ ^132^ ^]^ or Dirac source (DS) injection^[^ ^133^, ^134^ ^]^), in which the Boltzmann thermal distribution dominated limit of SS is no more a governing factor and newly established SS could be less than 60 mV dec^−1^ at room temperature. The devices with higher *g*_m_ and lower SS have sharp switching behavior from their off‐states to on‐states and vice versa and, therefore, could provide a large downscaling space of *V*_DD_ without sacrificing the off‐state performance.

## 4 High‐Performance SWCNT‐FET/TFT Electronics

### 4.1 Ballistic Transport in SWCNTs

Ballistic transport is highly demanded in high‐performance electronic devices as it yields high current driving capability and high operating speed. Like bulk semiconducting materials, the electrical transport in SWCNTs is also affected by scattering events induced by defects or lattice vibrations, giving rise to electrical resistance. However, due to its unique 1D nanostructure, SWCNT contacted with 3D metal electrodes exhibits a quantized resistance.^[^ ^135^, ^136^ ^]^ As discussed in Section 2, the motion of the electrons along the circumference of an SWCNT is confined because of the periodic boundary condition, leading to a series of discrete quantized states. When the SWCNT contacts with metal electrodes, these discrete quantized states in the SWCNT could superimpose on the continuous states of the electrodes, producing a quantized contact resistance *R*_Q_. Theoretical studies indicate that each metallic SWCNT has two conducting channels with the energies near the Fermi levels of the electrodes, and each of the channels contribute a conductance quantum *G*_0_ (*G*_0_ = 2*e* ^2^/*h*, where *h* is Planck's constant) to the total conductance.^[^ ^137^, ^138^, ^139^ ^]^ Therefore, a metallic SWCNT has a quantized contact resistance *R*_Q_ = 1/(2*G*_0_) = *h*/4*e* ^2^ = 6.45 kΩ .^[^ ^19^ ^]^

For an SWCNT with a high structural integrity, the electron transport scattering rate in the SWCNT is low so that electrons could even have a very long mean‐free‐path *λ*_MFP_ of the order of a micrometer.^[^ ^19^ ^]^ In other words, when the SWCNT channel length is scaled down to the sub‐micrometer range, for example, ≤ 100 nm, the ballistic transport can take place and the SWCNT can act as a good ballistic conductor. Si‐MOSFETs with ultrashort channel length usually suffer from poor device performance due to the short‐channel effects.^[^ ^2^ ^]^ However, in SWCNT‐FETs with very short channel length, the gate electrostatic control is significantly enhanced owing to the 1D ultrathin structures, making the SWCNT‐FETs effectively immune to the short‐channel effects.^[^ ^140^ ^]^ As the channel length decreases, the contact resistances between the SWCNTs channel and the S(D) electrodes, rather than the channel resistance, are dominant, and their adverse impact on the ballistic transport becomes more significant. Schottky‐contact SWCNT‐FETs usually exhibit high contact resistances and the on‐state current (*I*_ON_) is thus exponentially decreased with increasing the SB height.^[^ ^15^, ^113^, ^118^, ^141^ ^]^ Therefore, mitigating the SBs and realizing ohmic contact to SWCNTs with low contact resistances are crucial to achieve ballistic transport in high‐performance SWCNT‐FETs.^[^ ^142^ ^]^

### 4.2 Progress in High‐Performance SWCNT‐FETs

As discussed in Section 4.1, reducing the channel length and the contact resistance are two important routes to implement ballistic transport in SWCNTs. Considerable efforts on these two routes have been devoted to developing ballistic SWCNT‐FETs.

**Figure 6:**
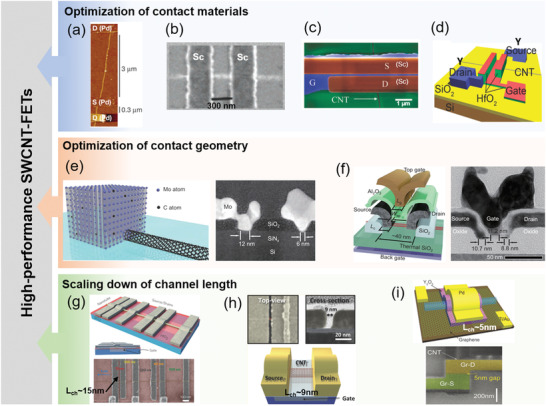
Development of high‐performance SWCNT‐FETs. a–d) Ohmic‐contacted SWCNT‐FETs with typical metal electrodes such as Pd, Sc, and Y. (a) Reproduced with permission.^[^ ^13^ ^]^ Copyright 2003, Springer Nature. (b) Reproduced with permission.^[^ ^24^ ^]^ Copyright 2008, American Chemical Society. (c) Reproduced with permission.^[^ ^26^ ^]^ Copyright 2007, American Chemical Society. (d) Reproduced with permission.^[^ ^143^ ^]^ Copyright 2009, American Chemical Society. e,f) SWCNT‐FETs with end‐bonded contact formed in a solid‐state carbide‐formation reaction.^[^ ^31^, ^39^ ^]^ (e) Reproduced with permission.^[^ ^31^ ^]^ Copyright 2017, The American Association for the Advancement of Science. (f) Reproduced with permission.^[^ ^39^ ^]^ Copyright 2015, The American Association for the Advancement of Science. g–i) Downscaling of the SWCNT channel length *L*_ch_ in SWCNT‐FETs. (g) Reproduced with permission.^[^ ^29^ ^]^ Copyright 2012, American Chemical Society. (h) Reproduced with permission.^[^ ^30^ ^]^ Copyright 2017, The American Association for the Advancement of Science. (i) Reproduced with permission.^[^ ^140^ ^]^ Copyright 2010, Springer Nature.

In 2003, Javey et al. successfully fabricated ohmic‐contact p‐type SWCNT‐FETs using the high work‐function metal Pd as the S/D electrodes,^[^ ^13^ ^]^ realizing zero or slightly negative SBs at the Pd/SWCNT contacts and room‐temperature ballistic transport in a short channel (*L*_ch_ = 300 nm) device (**Figure** **6**a) with on‐state conductance *G*_on_ ≈ 0.8*G*_0_. For n‐type ballistic devices, Zhang et al. studied the Sc‐contacted SWCNT‐FETs (Figure 6b) and found almost ballistic electron transport and metallic‐like behavior of the on‐state conductance (≈0.98*G*_0_ at 250 K),^[^ ^26^ ^]^ suggesting barrier‐free ohmic Sc/SWCNT contacts. By further optimizing the device with a self‐aligned top‐gated structure and adopting high‐*k* HfO_2_ as the dielectric layer,^[^ ^24^ ^]^ the Sc‐contacted n‐type SWCNT‐FET (*L*_ch_ = 120 nm) (Figure 6c) exhibited excellent on‐ and off‐state performance with *G*_on_ ≈ 0.64*G*_0_, *g*_m_ ≈ 25 µS, and SS ≈ 100 mV dec^−1^. In addition to Sc, the low work‐function metal Y (3.1 eV) can also form an ohmic contact with the conduction band of the SWCNT channel (Figure 6d).^[^ ^143^ ^]^ The Y‐contacted SWCNT‐FET (*L*_ch_ = 400 nm) exhibited near‐ballistic electron transport with a room temperature on‐state conductance of ≈1.1*G*_0_.

In a ballistic device, the channel resistance is negligible, and the contact resistance mainly determines the device performance. The most commonly used contact geometry in SWCNT‐FETs is side‐contact or planar‐contact. The contact resistance of such contact schemes increases rapidly with decreasing of the contact area and it is proportional to the contact length. Previous experiments have showed that the contact resistance increased from ≈5 kΩ with contact length more than 200 nm to ≈65 kΩ with contact length of 9 nm.^[^ ^140^ ^]^ The increase in the contact resistance has become a major performance roadblock to ultrasmall scaled devices. In 2015, Cao et al. proposed an end‐bonded contact geometry (Figure 6e), in which the ends of the SWCNT channel were attached to the deposited Mo electrodes through carbide bonds formed in a solid‐state carbide‐formation reaction.^[^ ^39^ ^]^ Owing to the formation of the carbide bonds, the end‐bonded contact showed a zero SB and a size‐independent contact resistance, resulting in an excellent downscaling behavior of the contact without increasing resistance. The Mo end‐bonded SWCNT‐FET (*L*_ch_ = 60 nm), with contact length scaled down to 9 nm, exhibited ballistic hole transport with a device resistance below 36 kΩ and an on‐state current of 15 µA. In 2017, they further reduced the channel length to 11 nm and constructed an end‐bonded device with ultrascaled contact length (≈10 nm) (Figure 6f).^[^ ^31^ ^]^ The entire device has been scaled down to a tiny footprint of 40 nm, and delivered a higher current density above 0.9 mA µm^−1^ at a low supply voltage of 0.5 V with an SS of 85 mV dec^−1^.

When the channel length is scaled down to the deep sub‐micrometer range, SWCNT‐FETs can still maintain their performance and are immune to the short‐channel effects. In 2010, Franklin and Chen investigated the scaling behavior of the Pd‐contacted SWCNT‐FETs with the channel length from 3 µm to 15 nm (Figure 6g).^[^ ^140^ ^]^ The 15 nm device exhibited room‐temperature conductance of 0.7*G*_0_ and peak transconductance of 40 µS. The nearly ballistic transport resulted in the device resistance of about 11 kΩ, which approaches the quantum limit resistance (*R*_Q_ = 6.45 kΩ). When the channel length was further reduced to 9 nm (Figure 6h),^[^ ^29^ ^]^ the device exhibited a diameter‐normalized current density of 2.41 mA µm^−1^, which was four times more than that of the Si nanowire devices with similar channel length. The contact resistance was extracted to be 6.6 kΩ, suggesting excellent contact properties at the Pd/SWCNT interface. In 2017, Qiu et al. developed a top‐gated Pd‐contacted SWCNT‐FETs with a 5 nm gate length, which delivered a large on‐state current of 20 µA and an ultrasmall device resistance of 10 kΩ at a low bias of 0.4 V.^[^ ^30^ ^]^ By introducing graphene as the S/D electrodes, the 5 nm graphene‐contacted device (Figure 6i) maintained the excellent on‐state performance with a much smaller SS of 73 mV dec^−1^, which was attributed to the improved gate efficiency induced by the ultrathin graphene S/D. Although there have been a few pioneering theoretical studies on sub‐5 nm gate‐length SWCNT‐FETs,^[^ ^144^, ^145^, ^146^ ^]^ accurate simulation and comparison with the existing experimental data were highly desirable. Recently, Xu et al. used the first‐principles quantum transport approach (benchmarked with the performance of 5 nm gate length SWCNT‐FETs^[^ ^30^ ^]^) to investigate the performance limit of sub‐5 nm gate‐length SWCNT‐FETs with a gate‐all‐around (GAA) device geometry.^[^ ^37^ ^]^ It was found that the device could be potentially scaled down to 2 nm gate‐length and fulfill high‐performance with an on‐state current of more than 1000 µA µm^−1^, an intrinsic delay time of 0.02 ps, and an SS of 150 mV dec^−1^. However, considering the tradeoff between the performance and power consumption, 5 nm gate‐length seemed to be the optimum scaled limit.

### 4.3 State‐of‐the‐Art of SWCNT‐TFTs and ICs

While a variety of individual SWCNT‐based FETs have been demonstrated with performance preceding Si‐based counterparts (with similar gate length) and even toward the theoretical limits,^[^ ^30^, ^31^ ^]^ the high‐performance and large‐scale IC applications require a high current drive capability and a high uniformity of device performance, which are unfeasible to be realized using an arbitrary individual SWCNT as the channel. To develop SWCNT‐based high‐performance IC electronics, a feasible channel can be constructed with a large number of SWCNTs in order to smooth out the arbitrary chirality, diameter, length, and orientation. SWCNT thin‐films in the form of either random network or aligned array have been extensively studied and utilized to build TFTs and ICs. As‐grown SWCNTs contain both semiconducting and metallic tubes and how to get rid of metallic ones is a challenge as the processes removing these metallic tubes unavoidably give rise to creation of defects in semiconducting tubes. To reduce the short‐circuit caused by metallic tubes across the S and D electrodes, the network‐channel length must be designed to be much larger than the average length of the SWCNTs. As a result, the tube–tube junctions and the defects in the network‐channel could severely affect the device performance, leading to a low carrier mobility and poor on‐state performance. The resulting ICs typically had an operating speed less than 1 MHz,^[^ ^41^, ^48^ ^]^ much less than that (approximately GHz) of conventional Si‐based ICs. Recently, the semiconducting purity of solution‐processed SWCNTs has been remarkably increased up to 99.99% through several dispersion and purification technologies.^[^ ^59^, ^68^, ^72^, ^73^ ^]^ Availability of a highly pure solution‐processed SWCNTs network makes high‐performance scalable SWNCT ICs feasible.^[^ ^147^, ^148^ ^]^ Thanks to the development of alignment techniques, such as dose‐controlled floating evaporative self‐assembly (DFES),^[^ ^75^ ^]^ dimension‐limited self‐alignment (DLSA),^[^ ^58^ ^]^ DNA‐assisted assembly,^[^ ^149^ ^]^ and Langmuir–Blodgett (LB)^[^ ^76^, ^77^ ^]^ or Langmuir–Schaefer (LS)^[^ ^78^ ^]^ method, a channel packed with a well‐aligned SWCNT array with a density of more than ≈100 tubes µm^−1^, a semiconducting purity of larger than 99.99%, and a relatively narrow distribution of the tube diameters could be utilized for high‐performance SWCNT‐based ICs.^[^ ^57^ ^]^

**Figure 7:**
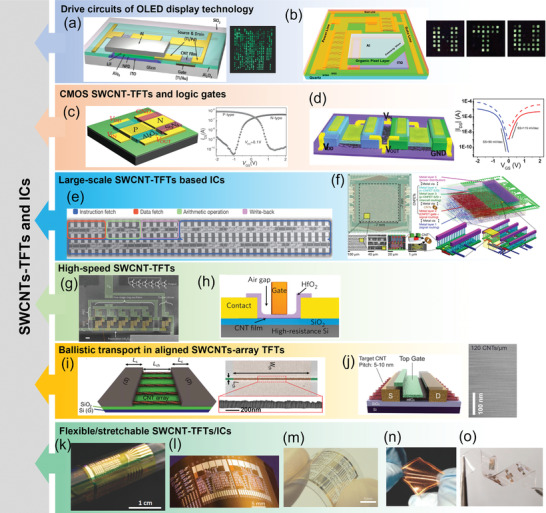
State‐of‐the‐art of SWCNT‐TFTs and ICs. a,b) SWCNT‐TFTs drive circuits for OLED display technology. (a) Reproduced with permission.^[^ ^44^ ^]^ Copyright 2011, American Chemical Society. (b) Reproduced under the terms of the Creative Commons CC BY license.^[^ ^45^ ^]^ Copyright 2015, Springer Nature. c,d) CMOS SWCNT‐TFTs and logic gates. (c) Reproduced with permission.^[^ ^49^ ^]^ Copyright 2013, Wiley‐VCH. (d) Reproduced with permission.^[^ ^54^ ^]^ Copyright 2017, American Chemical Society. e) SEM image of an SWCNT computer. Reproduced with permission.^[^ ^151^ ^]^ Copyright 2013, Springer Nature. f) Image and schematic 3D layout of a 16‐bit (RV16X‐NANO) microprocessor. Reproduced with permission.^[^ ^56^ ^]^ Copyright 2019, Springer Nature. g,h) High‐speed SWCNT‐TFTs and RO circuits. (g) Reproduced with permission.^[^ ^147^ ^]^ Copyright 2017, Springer Nature. (h) Reproduced with permission.^[^ ^152^ ^]^ Copyright 2018, Springer Nature. i,j) Ballistic aligned‐SWCNTs‐array TFTs. (i) Reproduced with permission.^[^ ^58^ ^]^ Copyright 2020, The American Association for the Advancement of Science. (j) Reproduced with permission.^[^ ^148^ ^]^ Copyright 2016, The American Association for the Advancement of Science. k–o) Flexible and stretchable SWCNT‐TFTs and ICs. (k) Reproduced with permission.^[^ ^41^ ^]^ Copyright 2008, Springer Nature. (l) Reproduced with permission.^[^ ^48^ ^]^ Copyright 2011, Springer Nature. (m) Reproduced with permission.^[^ ^157^ ^]^ Copyright 2012, American Chemical Society. (n) Reproduced with permission.^[^ ^161^ ^]^ Copyright 2013, Springer Nature. (o) Reproduced with permission.^[^ ^162^ ^]^ Copyright 2014, American Chemical Society.

As an important application, TFT has become the building block for back‐panel drive circuit of flat‐panel display technology. SWCNT‐TFTs drive circuits for active‐matrix (AM) organic light‐emitting diode (OLED) have been successfully demonstrated. In 2011, Zhang et al. reported a sorted s‐SWCNT‐TFTs drive circuit for an AMOLED display with 500 pixels (**Figure** **7**a).^[^ ^44^ ^]^ 348 out of 500 pixels can be turned on with a yield of ≈70%. In 2015, Zou et al. demonstrated, for the first time, static and dynamic AMOLED display driven by CVD‐grown SWCNT‐TFTs drive circuit (Figure 7b).^[^ ^45^ ^]^ The highly uniform TFTs performance ensured an excellent control capability of the drive circuit.

In addition to the drive circuits for flat‐panel displays, SWCNT‐TFTs have also been extensively used to construct digital ICs. Due to the lack of effective and stable approaches to fabricate n‐type SWCNT‐TFTs, the early demonstrated SWCNT‐based ICs were constructed only with p‐type SWCNTs network‐channel.^[^ ^41^, ^48^, ^150^ ^]^ In 2013, Gao et al. successfully fabricated p‐ and n‐type TFTs (Figure 7c) based on CVD‐grown SWCNTs network film using coating Al_2_O_3_ and Si_3_N_4_ thin layer on the channels, respectively.^[^ ^49^ ^]^ Both the p‐ and n‐type TFTs had comparable on‐ and off‐state performances, and the constructed CMOS ICs demonstrated logic functionalities. In 2017, Yang et al. demonstrated solution‐processed high purity (>99.9%) s‐SWCNTs complementary TFTs (Figure 7d)^[^ ^54^ ^]^ using a doping‐free technology^[^ ^26^ ^]^ in which Pd and Sc were used as the S/D electrodes to selectively inject holes and electrons into the channels to achieve p‐ and n‐type TFTs, respectively. The fabricated complementary TFTs (with a gate length of 1 µm) exhibited highly symmetrical electrical characteristics with significant improvement in the on‐state performance. A medium‐scale IC, 4‐bit full adders containing 132 complementary TFTs, were realized and functioned well with 100% yield.

Although SWCNT‐TFT technologies have developed for more than a decade, only very basic logic circuits have been demonstrated. In 2013, the first CNT computer (Figure 7e) was successfully demonstrated using 178 SWCNT‐TFTs.^[^ ^151^ ^]^ More than 99.99% of the metallic tubes in the CVD‐grown aligned SWCNTs array were removed by electrical burn‐off process. The CNT computer can execute multiple programs synchronously. As a major advance for very‐large‐scale SWCNT‐based integrated system, a 16‐bit microprocessor (RV16X‐NANO) (Figure 7f) consisting of more than 14 000 CMOS SWCNT‐TFTs was developed by MIT in 2019.^[^ ^56^ ^]^ Solution‐sorted s‐SWCNTs (99.99% purity) network was used in the microprocessor, and optimized processing and circuit design techniques were employed to overcome the inherent SWCNT problems, such as metallic tubes and tube‐bundles. This 16‐bit microprocessor could execute standard 32‐bit instructions on 16‐bit data and addresses.

As discussed above, SWCNT‐TFTs with ultrashort channels are contact dominated with the quasi‐ballistic transport mechanism and different from that of junction‐dominated long‐channel SWCNT‐TFTs. The operation speed of SWCNT‐based ICs has been significantly promoted with downscaling of the channel length into the sub‐micrometer range.^[^ ^147^, ^152^, ^153^, ^154^ ^]^ In 2017, Han et al. reported a five‐stage CMOS ring oscillator (RO) constructed by solution‐processed, self‐assembled s‐SWCNTs (purity >99.9%) array TFTs with channel length of 100 nm (Figure 7g).^[^ ^147^ ^]^ The RO circuit had an oscillation frequency of 282 MHz and a corresponding sub‐nanosecond stage delay of 355 ps at *V*_DD_ of 1.9 V. A remarkable leap in the operation speed (up to GHz regime) of SWCNT‐based ICs was achieved by Zhong et al. in 2018.^[^ ^152^ ^]^ They fabricated top‐gated p‐type devices using solution‐processed s‐SWCNTs (purity >99.99%) network film. Their devices were optimized with a 120 nm thick gate metal to suppress the gate series resistance and 70 nm air gaps between the G and S/D contacts to reduce the parasitic capacitances (Figure 7h). The constructed five‐stage RO with a gate length of 115 nm yielded an oscillation frequency up to 5.54 GHz with an average gate delay of 18 ps, which was comparable to that of Si CMOS ICs with a gate length of 130 nm.^[^ ^155^ ^]^

Although ballistic transport has been realized in the FETs containing an individual SWCNT in the channel, it is difficult to achieve ballistic transport in a SWCNTs‐network‐film channel even when the channel length is scaled down to the sub‐micrometer scale as the intertube junctions and random tube orientations in the network greatly deteriorate carrier mobility and on‐state conductance. In comparison, a well‐aligned s‐SWCNT array can be an ideal ballistic‐transport channel material if excellent SWCNT alignment, high packing density, and high semiconducting purity can be obtainable. In 2016, Brady et al. reported quasi‐ballistic transport in a solution‐processed aligned s‐SWCNTs (purity >99.98%) array (Figure 7i).^[^ ^148^ ^]^ A “rinsed and annealed” post‐treatment process and an optimized tube density (47 tubes µm^−1^) were adopted to improve the contact properties and reduce the electrostatic screening effect arising from intertube interactions, leading to an excellent on‐state performance with the conductance as high as 0.46*G*_0_ per tube, nearly seven times higher than that of previously reported SWCNTs‐array‐based devices. The saturated on‐state current density (392 µA µm^−1^) of the device (*L*_ch_ = 140 nm) was 1.9‐fold higher than that of a 90 nm node Si MOSFET. Recently, a significant breakthrough in producing aligned s‐SWCNTs array has been achieved by Liu et al.^[^ ^58^ ^]^ They developed a multiple‐dispersion sorting process and DLSA method to successfully assemble aligned s‐SWCNTs array with extremely high purity (≈99.9999%), adjustable tube‐pitch (5–10 nm), narrow diameter distribution (≈1.5 nm), and full wafer coverage (4 in.). A top‐gated SWCNT‐array TFT with an optimal tube density of 120 tubes µm^−1^ in the channel (Figure 7j) exhibited an on‐state current density of 1.3 mA µm^−1^ and a peak transconductance of 0.9 mS µm^−1^ at *V*_DS_ = 1 V. Both values were higher than that of Si‐based MOSFETs with a similar gate length (≈100 nm). In addition, a five‐stage RO circuit constructed by the SWCNT‐array TFTs was able to work at an oscillation frequency of 8.06 GHz under *V*_DD_ = 2.6 V. However, due to the large interface trapped charge density (≈10^12^ cm^−2^),^[^ ^156^ ^]^ the SS of the device is only 190 mV dec^−1^, much below the requirement (<100 mV dec^−1^) for digital ICs.

In addition, the excellent electronic and mechanical properties have made SWCNT thin‐films especially suitable for flexible and stretchable electronics. There have already been lots of demonstrations on SWCNT thin‐film‐based flexible/stretchable electronic devices.^[^ ^41^, ^48^, ^157^, ^158^, ^159^, ^160^, ^161^, ^162^, ^163^, ^164^ ^]^ Using high‐purity s‐SWCNTs solution, Wang et al. fabricated SWCNT‐TFTs and fundamental logic gates on an ultrathin polyimide substrate (Figure 7k).^[^ ^157^ ^]^ The flexible SWCNT‐TFTs with channel length of 4 µm exhibited highly uniform device performance with on‐state current and transconductance up to 15 µA µm^−1^ and 4 µS µm^−1^, respectively. Additionally, such highly flexible devices and logic gates exhibited excellent stability even after 1000 bending cycles. High‐performance flexible TFTs and ICs have also been demonstrated using CVD‐grown SWCNT networks. Cao et al. adopted stripe‐patterning to reduce the percolating metallic pathways in the SWCNT networks.^[^ ^41^ ^]^ The fabricated transistors showed *I*_ON_/*I*_OFF_ as high as 10^5^ and carrier mobility of 80 cm^2^ V^−1^ s^−1^. A 4‐bit row decoder circuit (Figure 7l) consisting of 88 transistors was further demonstrated on a plastic substrate. By controlling the percolation threshold density of the SWCNT networks, Sun et al. fabricated TFTs and ICs on flexible and transparent substrates (Figure 7m) with high *I*_ON_/*I*_OFF_ of 10^6^ and field‐effect mobility of 35 cm^2^ V^−1^ s^−1^, respectively.^[^ ^48^ ^]^ Because of the large length/diameter ratio, SWCNTs are naturally highly curved and entangled in their thin‐film form, making them promising materials for stretchable electronics.^[^ ^165^, ^166^ ^]^ Chae et al. reported a highly stretchable TFT (Figure 7n) with graphene electrodes, SWCNT network channel, and a geometrically wrinkled Al_2_O_3_ dielectric layer.^[^ ^161^ ^]^ The randomly distributed wrinkles imparted the Al_2_O_3_ layer a certain degree of stretchability. The resulting devices retained performance after stretching and releasing for over 1000 cycles under a tensile strain up to 20%. Using intrinsically stretchable dielectric materials such as ion gel, Xu et al. fabricated stretchable SWCNT‐TFTs (Figure 7o) that could be stretched up to 50%.^[^ ^162^ ^]^ There was no appreciable degradation in the device performance after repeated mechanical cycling. **Table** **2** summarizes the device structures and performances of the state‐of‐the‐art SWCNT‐FETs/TFTs.

**Table 2:** Device structures and performances of state‐of‐the‐art SWCNT‐FETs/TFTs. (BG: back‐gated; TG: top‐gated; *G*_0_: conductance quantum (2*e* ^2^/*h*))

Refs.	Device structure	Channel	S/D	Gate dielectric	Channel length [nm]	*I*_ON_ [mA µm^−1^]	*I*_ON_/*I*_OFF_	*G*_on_	SS [mV dec^−1^]	*g*_m_
[13]	BG	Single tube	Pd	500 nm SiO_2_	300	7	10^6^	0.8*G*_0_	170	
[26]	BG	Single tube	Sc	100 nm SiO_2_	300	10	10^5^	0.98*G*_0_	250	
[24]	TG	Single tube	Sc	15 nm HfO_2_	120	5	10^4^	0.64*G*_0_	100	25 µS per tube
[143]	BG	Single tube	Y	500 nm SiO_2_	400	12	10^5^	1.1*G*_0_	400	
[39]	BG	Single tube	Mo	20 nm SiN*_x_*	60	9	10^7^		100	
[31]	TG	Single tube	Co–Mo	5 nm Al_2_O_3_	11	0.9	10^3^		85	
[140]	BG	Single tube	Pd	10 nm HfO_2_	15		10^5^	0.7*G*_0_	85	40 µS per tube
[29]	BG	Single tube	Pd	3 nm HfO_2_	9	2.41	10^4^		94	55 µS per tube
[30]	TG	Single tube	Pd	3.5 nm HfO_2_	5	15	10^4^	0.64*G*_0_	105–130	
[54]	TG	Network	Pd/Sc	18 nm HfO_2_	1000	0.015	10^5^		73–82	20 µS µm^−1^
[157]	BG	Network	Pd	20 nm Al_2_O_3_	4000	0.015	400			4 µS µm^−1^
[147]	BG	Network	Pd/Sc	4 nm Al_2_O_3_	100		10^5^			
[152]	TG	Network	Pd	7.5 nm HfO_2_	120	0.55	10^5^		160	460 µS µm^−1^
[154]	TG	Array	Pd	5 nm HfO_2_	95	1.92				1400 µS µm^−1^
[148]	BG	Array	Pd	15 nm SiO_2_	140	0.392	10^4^	0.46*G*_0_ per tube		100 µS µm^−1^
[58]	TG	Array	Pd	7.3 nm HfO_2_	120	1.3	10^5^		190	900 µS µm^−1^

## 5 Low‐Power SWCNT‐FET/TFT Electronics

**Figure 8:**
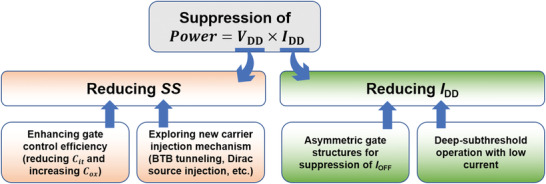
Two main strategies for suppression of the power consumption of SWCNT‐FETs/TFTs: decrease of the SS and reduction of the off‐state leakage current.

With downscaling of the feature size of Si MOSFETs, the ICs are of a high operation speed and high driving capability. Unfortunately, the static power consumption was not reduced, but, instead, increased exponentially and became a bottleneck for further development of ICs.^[^ ^118^, ^167^, ^168^ ^]^ The device operation with ultralow‐voltage (<0.5 V) and ultralow‐power (<1 nW) is highly demanded in the emerging electronic applications such as portable and wearable systems because of the limited battery lifetime or battery‐less energy harvesting strategy. SWCNT‐FET‐based ICs with high speeds and high driving capabilities usually required a large supply voltage (1–2 V) to guarantee a high operation speed and correct logical functions. ^[^ ^54^, ^55^, ^58^, ^152^ ^]^ This leads to high either static or dynamic power consumption. Also, a small bandgap (below 1 eV) of SWCNTs usually results in an ambipolar conduction behavior, high off‐state leakage current, and degraded SS. All these poor off‐state characteristics cause outrageously high static power consumption and eventually restrict the low‐power electronic applications of SWCNT‐FETs/TFTs. Therefore, two main strategies for suppression of the power consumption (**Figure** **8**) have been attempted. One is to reduce the SS. As discussed in Section 3.3, a small SS implies a large downscaling range of the supply voltage *V*_DD_, resulting in reduction in the device power consumption. To achieve this goal, many efforts have been devoted to enhancing gate control efficiency and exploring new carrier injection mechanisms (e.g., BTB tunneling^[^ ^132^, ^169^ ^]^ and Dirac source injection^[^ ^133^, ^134^ ^]^). The other strategy is to suppress off‐state leakage current. Several device structures that can inhibit ambipolar behavior and off‐state leakage current have been proposed and implemented.^[^ ^105^, ^106^, ^107^, ^108^, ^109^, ^110^, ^111^ ^]^ In addition, the devices and ICs working in deep‐subthreshold region with small current have also been reported.^[^ ^170^ ^]^ In this section, these two strategies will be discussed in detail.

### 5.1 Effective Reduction of SS

From discussion on expression of SS in Section 3.3, lowering the interface trapped charge density (i.e., reducing *C*_it_) and enhancing the gate efficiency (i.e., increasing *C*_ox_) are the most straightforward measures to reduce SS toward its room temperature limit, ≈60 mV dec^−1^ governed by the Boltzmann thermal equilibrium distribution. The density of trapped charges and adsorbates on the gate dielectric layer could be reduced through a dehydration reaction occurred in a hexamethyldisilazane (HMDS) vapor prime process, leading to a small SS of 62 mV dec^−1^ and an average lowest static power consumption of ≈10 pW in a SWCNT‐TFT‐based inverter.^[^ ^163^ ^]^ However, in solution‐processed SWCNTs, the surfactant/polymer residues around the SWCNTs usually induce high interface trapped charge density up to 10^12^ cm^−2^,^[^ ^156^ ^]^ which are hardly removed using simple rinsing and annealing processes.^[^ ^58^ ^]^

**Figure 9:**
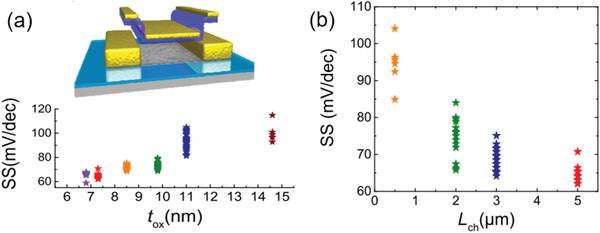
Downscaling behavior of self‐aligned top‐gated SWCNTs‐network‐TFTs. a) Schematic device structure and downscaling behavior (statistical SS distribution as a function of *t*_ox_) of the devices with a channel length of 5 µm at *V*_DS_ = −0.1 V. b) Lateral downscaling behavior (statistical SS distribution as a function of channel length) of the devices with *t*_ox_ = 7.3 nm at *V*_DS_ = −0.1 V. Reproduced with permission.^[^ ^171^ ^]^ Copyright 2018, AIP Publishing.

Since the thickness of gate dielectric is of great importance in determining gate capacitance and gate control efficiency (see Section 3.3), the exploration of the downscaling behavior and the scaling limit of the gate dielectric thickness could provide a benchmark for selection of dielectric materials and their thickness. Reducing gate dielectric thickness and engaging high‐*k* dielectric materials have been evidenced to be efficient for reduction of the SS. Zhao et al. fabricated self‐aligned top‐gated s‐SWCNTs‐network‐TFTs with downscaled high‐*k* gate dielectric (HfO_2_) thickness *t*_ox_ .^[^ ^171^ ^]^ Their devices exhibited a clear trend of the SS decreasing with downscaling of *t*_ox_ (**Figure** **9**a). When *t*_ox_ was decreased to 7.3 nm, the average SS approached 62 mV dec^−1^. Unfortunately, the gate leakage current was found to increase apparently when *t*_ox_ was less than 6.8 nm. As a result, optimization of *t*_ox_ should be based on a trade‐off between a large leakage current and a small SS, between 7 and 10 nm for a HfO_2_ gate dielectric layer. In addition, the SS was found to be increased with downscaling of the channel length (Figure 9b). When the channel length is scaled down to deep‐sub‐micrometer, the most of SWCNTs in the channel could bridge the S and D directly. The fluctuations in SWCNTs orientations and diameters inevitably cause variation in *V*_TH_ of these tubes.^[^ ^171^, ^172^ ^]^ As a result, all these individual tubes could not concurrently switch off, incurring a poor SS.

**Figure 10:**
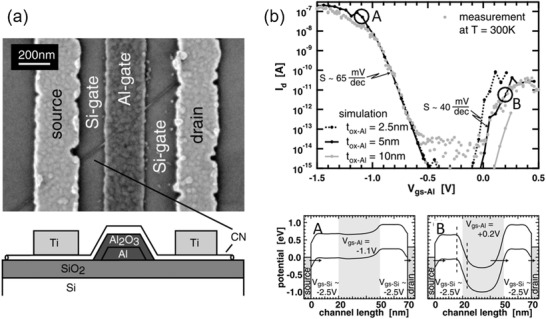
BTB tunneling in an individual SWCNT‐FET. a) SEM image and schematic cross‐sectional structure of a BTB tunneling SWCNT‐FET. b) Transfer characteristics of the device at *V*_DS_ = −0.5 V and *V*_gs‐Si_ = −3 V, and the corresponding energy band diagrams (in the lower panel) of the device for the “A” and “B” branches of the transfer characteristics, respectively. Reproduced with permission.^[^ ^132^ ^]^ Copyright 2004, American Physical Society.

To achieve an SS less than the room temperature limit, ≈60 mV dec^−1^, and also maintain a large on/off current ratio (*I*_ON_/*I*_OFF_ ≥ 10^4^) require a completely different carrier injection mechanism, in which the Boltzmann thermal distribution dominated injection is no more a dominant carrier injection mechanism. BTB tunneling^[^ ^173^, ^174^ ^]^ dominated injection has been found to be capable of an SS of ≈40 mV dec^−1^ at room temperature in an individual SWCNT‐FET (**Figure** **10**a).^[^ ^132^ ^]^ A Si back‐gate was employed to adjust the electrostatic potential of the nanotube channel close to the Fermi level of the S and D, while an Al gate could control the potential of the middle part of the nanotube channel, creating a p–n–p band structure. The device exhibited two conduction branches (Figure 10b). The “A” branch corresponds to thermionic emission dominated hole injection (see the energy band diagram for the “A” branch in Figure 10b) with an SS (≈65 mV dec^−1^) close to the limit at room temperature. Interestingly, the “B” branch presents an SS of ≈40 mV dec^−1^, which can be attributed to the bandpass‐filter‐like BTB tunneling. The tunneling occurs in the gate voltage window determined by the difference between the valence band near the source and the conduction band in the middle part of the channel (see the energy band diagram for the “B” branch in Figure 10b). The high‐energy tail of the holes injected from the source is cut off, resulting in a “cold” electronic system.^[^ ^123^, ^175^ ^]^ The device is analogous to a conventional MOSFET operating at a temperature below 300 K, but enabling an SS of less than 60 mV dec^−1^. Occurrence of the BTB tunneling in SWCNT‐FETs benefits from the ultrathin tunneling barrier that originates from the 1D nature of the nanotube channel and the excellent gate coupling.^[^ ^119^, ^132^ ^]^

**Figure 11:**
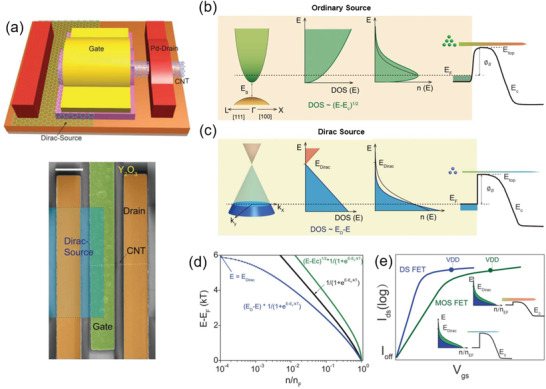
Graphene‐DS‐SWCNT‐FET. a) Schematic structure and SEM image of a typical graphene‐DS‐SWCNT‐FET (scale bar: 500 nm).^[^ ^133^ ^]^ Schematic energy dispersion, density of states (DOS), electron density distribution, and subexponentially decaying of thermally activated electron injection over a bulk channel barrier for b) an ordinary‐source and c) a Dirac‐source with superexponentially decaying of electron density at off‐state. d) Electron density distributions around the Fermi level for 3D (green curve) and 2D (black curve) semiconductor ordinary‐source and a graphene Dirac‐source (blue curve). e) Schematic transfer curves of an FET with an ordinary‐source (green curve) and a Dirac‐source (blue curve). Inset: Electron density distributions for an ordinary‐source (green) and a Dirac‐source (blue), and the thermally activated electron emission over the barrier at on‐state (upper part) and off‐state (lower part). Reproduced with permission.^[^ ^133^ ^]^ Copyright 2018, The American Association for the Advancement of Science.

In addition to the BTB tunneling devices, a novel steep‐slope device, called DS FET (**Figure** **11**a), was proposed by Qiu et al. in 2018.^[^ ^133^ ^]^ An individual s‐SWCNT channel was contacted to the n‐doped graphene source and Pd‐drain electrode. An ultrathin Y_2_O_3_ gate dielectric layer (equivalent oxide thickness (EOT) of 1.5 nm) provided strong gate coupling to the SWCNT channel. The DS‐SWCNT‐FET could function as an energy‐efficient electronic switch with an SS of 35 mV dec^−1^ at room temperature, a large *I*_ON_/*I*_OFF_ ≥ 10^6^, and a high *I*_ON_ of 6.5 µA at *V*_DS_ = −0.5 V. It is well known that in an ordinary Si‐based MOSFET, the DOS in the conduction band increases with energy, and the electron density *n*(*E*) subexponentially decays toward higher energy due to the Fermi distribution function (Figure 11b). In sharp contrast, graphene is of a linear energy dispersion near the Dirac points, which causes superexponentially decaying of electron density toward the Dirac points (Figure 11c), leading to a more localized electron density distribution around the Fermi energy *E*_F_ (blue curve in Figure 11d). The SS in a DS‐FET can be expressed as^[^ ^133^ ^]^

(7) $$\mathrm{SS}\;=\frac{1}{\xi }\left(1-\frac{k_{B}T}{E_{\mathrm{Dirac}}-\Phi_{B}}\right)\frac{k_{B}T}{e}\ln10\;$$

where Φ_B_ is the SB height at the off‐state; *E*_Dirac_ is the energy of the Dirac point; *ξ* = −dΦ_B_/d(*eV*_GS_) ≤ 1 is the gate efficiency that describes the sensitivity of the SB lowering to *V*_GS_. In a DS‐FET with the condition of *E*_Dirac_ − Φ_B_ > *nk*_B_ *T*, the SS could be smaller than (*k*_B_ *T*/*e*)ln10≅60 mV dec^−1^ at room temperature. The source acts as a “cold source” for the electronic system without a high energy tail of electron density distribution. Therefore, at the off‐state of the DS‐FET, the thermally activated electron density with energy over the SB decreases superexponentially with decreasing of *V*_GS_, resulting in a steeper SS compared with that in a conventional MOSFET (Figure 11e).

### 5.2 Suppression of Off‐State Leakage Current

SWCNT‐FETs typically suffer from a large off‐state leakage current, *I*_OFF_, because of the small bandgaps (0.3–0.6 eV) of SWCNTs.^[^ ^15^, ^24^, ^113^ ^]^ In this case, a high *I*_OFF_ is caused by carrier tunneling through the thin potential barrier formed by the small bandgap at the drain contact, in particular under a high gate control efficiency. The poor off‐state properties would inevitably lead to a significant increase in the static power consumption of SWCNT‐based ICs. In conventional Si MOSFETs, introducing a lightly doped region between the heavily doped drain region and the channel has been proven to be able to effectively suppress *I*_OFF_.^[^ ^176^, ^177^ ^]^ However, due to the lack of simple and efficient doping methods for SWCNTs, the *I*_OFF_ of SWCNT‐FETs is not easily suppressed.

**Figure 12:**
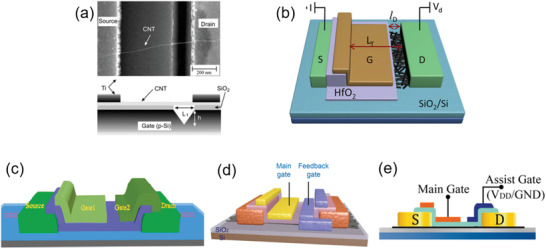
Asymmetric gate configurations for suppression of the off‐state leakage current. Asymmetric single‐gate structures with a) a trench or b) a gap between the gate and the drain electrodes. (a) Reproduced with permission.^[^ ^105^ ^]^ Copyright 2020, Elsevier. (b) Reproduced with permission.^[^ ^110^ ^]^ Copyright 2004, American Chemical Society. Asymmetric dual‐gate structures with c,d) specially biased partial‐gate or e) assistant gate. (c) Reproduced with permission.^[^ ^106^ ^]^ Copyright 2015, American Chemical Society. (d) Reproduced with permission.^[^ ^108^ ^]^ Copyright 2019, Springer Nature. (e) Reproduced with permission.^[^ ^109^ ^]^ Copyright 2020, American Chemical Society.

Recently, several kinds of asymmetric device configurations (e.g., partial‐gate and dual‐gate structures) have been reported to effectively suppress ambipolarity and *I*_OFF_ in SWCNT‐FETs/TFTs.^[^ ^105^, ^106^, ^107^, ^108^, ^109^, ^110^ ^]^ In the common asymmetric single‐gate structure (**Figure** **12**a ^[^ ^110^ ^]^ and Figure 12b;^[^ ^105^ ^]^ a similar design also shown in Figure 3b), a trench or a gap between the gate and the drain electrode is introduced. In consequence, the gate has a less impact on the energy band near the drain contact so that a thick barrier forms near the drain contact at the off‐state (see Figure 3e) to efficiently suppress the electron tunneling current (*I*_OFF_) even at a high *V*_DS_.

In addition to asymmetric single‐gate structures, specially designed dual‐gate structures have also been adopted to reduce *I*_OFF_. Qiu et al. fabricated a dual‐gate FET (Figure 12c) based on a single SWCNT channel,^[^ ^106^ ^]^ where the main‐gate (Gate1, overlapped with the source) covers most of the channel and controls the electrostatic potential, and the extra‐gate (Gate2, overlapped with the drain) directly connects to the drain electrode and makes the drain contact region extend into the channel (similar to the structure shown in Figure 3c). In this design, Gate2 does not function as an ordinary gate. Instead, it actually extends the drain potential, shielding the electrical potential from Gate1 away from the SWCNT/drain contact. As a result, a thick potential barrier near the drain can be obtained for remarkable suppression of *I*_OFF_. In 2019, Liu et al. adopted the dual‐gate structure in a SWCNT‐TFTs with a channel length of 375 nm (Figure 12d).^[^ ^108^ ^]^ Compared to a self‐aligned symmetric single‐gate device, the dual‐gate device exhibited a significantly suppressed *I*_OFF_ by 2 orders of magnitude. Recently, Zhao et al. reported CMOS SWCNT‐TFTs with a dual‐gate structure (Figure 12e),^[^ ^109^ ^]^ but different configuration from that in Figure 12d. In addition to the main‐gate overlapped with the source, an assistant gate was introduced and overlapped with the drain contact. The assistant gates in p‐ and n‐type TFTs were biased to the ground and *V*_DD_, respectively, to maintain a thick barrier at the drain contact and suppress the carrier tunneling at the off‐state. The SWCNT CMOS logic gates constructed using the dual‐gate devices demonstrated suppressed static power dissipation by 3 orders of magnitude compared to the SWCNT CMOS circuits built on symmetric single‐gate devices.

Exploring the capability of FETs/TFTs in the deep‐subthreshold regime (i.e., near the off‐state) is an effective approach to realization of ultralow‐power electronics because of low subthreshold current and steep SS. Oxide semiconductor FETs^[^ ^178^ ^]^ as well as organic semiconductor FETs^[^ ^179^, ^180^, ^181^ ^]^ have been shown excellent deep‐subthreshold characteristics with a power less than 1 nW. Very recently, Portilla et al. have reported deep‐subthreshold SWCNT‐TFTs and ICs^[^ ^170^ ^]^ whose static power consumption was only 1 pW at a supply voltage of 0.2 V. A self‐assembled monolayer (SAM) was incorporated together with an ultrathin (≈3 nm) AlO*_x_* layer to form a high quality hybrid AlO*_x_*/SAM gate dielectric. The resultant flat‐band voltage was in favor of a well‐balanced ambipolar conductance in the deep‐subthreshold regime, leading to ultralow‐power consumption of their SWCNT‐based ICs. In addition, Zou et al. recently fabricated SB‐contacted SWCNT‐TFTs that were operated in the subthreshold region.^[^ ^182^ ^]^ A thin high‐*k* gate dielectric and asymmetric gate configuration enabled highly efficient gate modulation of the SB at the source contact, resulting in excellent subthreshold switching characteristics with small SS (≈67 mV dec^−1^), large current on/off ratio (≈10^6^), and low off‐state current (≈0.5 pA). The p‐channel metal–oxide–semiconductor (PMOS) inverter built with the subthreshold SB‐SWCNT‐TFTs presented almost rail‐to‐rail outputs with ultralow power consumption less than 1 pW under a small supply voltage of 0.2 V.

## 6 Outlook

Over the past two decades, remarkable achievements have been made in both fundamental research and applications of SWCNT‐based electronics. SWCNT thin‐films have become highly promising for unique electronic applications, including flexible electronics, large‐scale ICs, and sensors. It has been theoretically predicted that computers based on SWCNT‐FETs/TFTs can provide a power‐performance improvement of ten times over computers based on Si‐CMOS technology.^[^ ^16^ ^]^ Compared to the TFTs based on other semiconductors, like amorphous silicon, polysilicon, and organic semiconductors, SWCNT‐TFTs offer significantly better performance. SWCNT‐FETs/TFTs and ICs have been demonstrated with comparable or even supercharacteristics in comparison of conventional Si‐based counterparts with similar gate lengths.^[^ ^30^, ^54^, ^58^, ^152^ ^]^ All these advancements have revealed a great potential of SWCNTs for future electronic applications in the more Moore era. To that destination, several key challenges must be overcome before commercialization of SWCNT‐based electronics comes into reality.

From the perspective of large‐scale ICs fabrication, SWCNTs should be well aligned with a density of 100–200 tubes µm^−1^ in the wafer scale. High semiconducting purity (>99.99%) and a narrow chirality distribution are required to guarantee a high uniformity of SWCNT‐based devices and ICs. Recent advances in SWCNTs dispersion, sorting, and self‐assembly alignment have made SWNCTs toward the requirements. Recently, 200 mm wafer‐scale uniformity and reproducibility of SWCNT‐TFTs fabricated using commercial Si manufacturing facilities have been achieved.^[^ ^80^ ^]^ Along the development, efforts are still required to mitigate the effects of defects, interface trapped charges, and polymer residues around the SWCNTs.

In addition, the SWCNT‐based device design and fabrication processes are still far from mature compared to that of modern Si‐based electronics. New device structures and fabrication techniques are to be explored to reduce the parasitic capacitance between the G and the S/D and the contact resistance between the SWCNTs channel and the S/D in order to raise the operating speed and reduce the power consumption of the SWCNT‐based devices and ICs.

SWCNTs, with a dangling‐bond‐free quasi‐1D carbon sp^2^ atomic architecture, are of a unique distribution of electronic states, which is distinct from other semiconductors. Novel device designs as well as the electrode materials are required to reduce the contact resistance between SWCNTs and S/D pads, mitigate the impact of the small bandgap on the electron transport in the deep‐subthreshold regime, and enhance the gate control efficiency. Compared to the common planar packaging technology adopted in SWCNT‐FETs/TFTs and ICs at present, 3D integrated SWCNT‐based ICs using multilayer interconnects could further boost the device operation speed and cut down the device operation power.^[^ ^183^ ^]^

## Conflict of Interest

The authors declare no conflict of interest.
